# A Recommendation Approach for Rating Prediction Based on User Interest and Trust Value

**DOI:** 10.1155/2021/6677920

**Published:** 2021-03-06

**Authors:** Hailong Chen, Haijiao Sun, Miao Cheng, Wuyue Yan

**Affiliations:** Department of Computer Science and Technology, Harbin University of Science and Technology, Harbin, Heilongjiang 150000, China

## Abstract

Collaborative filtering recommendation algorithm is one of the most researched and widely used recommendation algorithms in personalized recommendation systems. Aiming at the problem of data sparsity existing in the traditional collaborative filtering recommendation algorithm, which leads to inaccurate recommendation accuracy and low recommendation efficiency, an improved collaborative filtering algorithm is proposed in this paper. The algorithm is improved in the following three aspects: firstly, considering that the traditional scoring similarity calculation excessively relies on the common scoring items, the Bhattacharyya similarity calculation is introduced into the traditional calculation formula; secondly, the trust weight is added to accurately calculate the direct trust value and the trust transfer mechanism is introduced to calculate the indirect trust value between users; finally, the user similarity and user trust are integrated, and the prediction result is generated by the trust weighting method. Experiments show that the proposed algorithm can effectively improve the prediction accuracy of recommendations.

## 1. Introduction

The development of information technology and Internet makes human life more convenient and colorful, which also makes human life increasingly dependent on this, and the information data on the Internet also increases explosively. In the long run, it has become increasingly difficult to obtain effective information from a large amount of information, and mankind has gradually entered an era of information overload. A recommendation system is proposed to solve this problem. It aims to provide users with information or items (such as music, movies, and books) that may be of interest to users, to solve the problems caused by information overload. Successfully used in e-commerce and academic research [1]. There are many classifications of recommendation systems, such as content-based recommendation [2] and knowledge-based recommendation [3] and collaborative filtering. Collaborative filtering is the most prominent and commonly used recommendation technology [4, 5]. With the widespread use of recommendation algorithms, the problems of collaborative filtering technology have gradually emerged. The common problems include cold start [6, 7] and data sparsity [8–10].

To obtain as much useful information as possible to predict user preferences, some researchers have begun to use implicit feedback information [11–16]. Liu et al. [17] proposed a novel user similarity calculation method, which not only uses the rating data of text information but also includes the preferences of registered users, which improves the accuracy of recommendation and can alleviate the problem of data sparsity. As an important feature information in social information, the degree of attention between users provides a new basis for system recommendation [18]. Compared with rating information, social information can play a complementary role, but the direct social relationship is also very sparse, just like user-item rating. Reference [19] improved the recommendation efficiency of collaborative filtering by using sparse evaluation data and sparse social data provided by users. In [20], probability matrix decomposition is used to predict the score, and social trust is added in the construction of the user feature model. This method can improve the recommendation accuracy in the case of sparse data. TrustSVD model [21] integrates explicit and implicit information of user rating and user trust. Experimental results show that TrustSVD is superior to several baseline methods of relative ratio. Guo et al. [22] proposed a scoring prediction framework, which can use trusted and distrusted networks to achieve the purpose of accurate prediction.

Based on the trust propagation mechanism in social networks, we can predict the trust degree of unconnected users and infer the indirect trust relationship between users. Especially when users do not have enough direct relationships, the problem of data sparsity can be effectively alleviated. In addition to trusted users that can affect the target user's choice of items, the user's own interest in the items also accounts for a large proportion. Based on this, this paper proposes a collaborative filtering recommendation algorithm which combines user interest and trust. First of all, the Bhattacharyya similarity is introduced into the traditional similarity calculation method; secondly, the direct trust degree and indirect trust degree of users are improved and integrated into the comprehensive trust degree. Finally, the user similarity and user trust are integrated, and the prediction results are generated by the trust weighting method. In summary, the main contributions of this paper are summarized as follows:

1. To solve the problem of dependence of traditional calculation methods on common scoring items, the Bhattacharyya similarity is applied to traditional calculation methods.
2. We introduce trust weight to improve the calculation of direct trust, and according to the trust transmission mechanism and the six-degree separation theory, the indirect similarity calculation is improved and integrated into the user's trust degree.
3. This paper proposes a hybrid recommendation algorithm which combines user interest and trust, which effectively alleviates the problem of data sparsity in collaborative filtering recommendation system.

## 2. Related Work

In this section, we briefly review some related studies about collaborative filtering (CF) and trust-based CF recommender systems.

### 2.1. Collaborative Filtering Recommendation Algorithm

Collaborative filtering recommendation algorithm is a widely used recommendation algorithm, which generates personalized recommendations by mining user interests. However, the collaborative filtering recommendation algorithm also has some shortcomings, such as cold start, data sparseness, and scalability. In response to the problem of data sparseness, researchers have made many attempts to improve collaborative filtering. Authors in [23] proposed a method of missing value filling, using certain technical methods to estimate the score items that do not exist in the score matrix and increase the number of score items in the score matrix to reduce the sparseness of the matrix. Salehi et al. proposed a recommendation algorithm that integrates item attributes for optimization [24]. In the algorithm, genetic algorithm is used to further optimize the potential attributes, and the weighted C-means algorithm is introduced to cluster users. Experimental results prove that the recommendation effect of the proposed algorithm is good and can also solve the scalability problem of the algorithm. The group recommendation algorithm proposed by Ghazarian et al. improves the similarity of items on the basis of the original technology, and the specific implementation method is to incorporate support vector machine technology [25]. Experiments show that this algorithm strengthens memory-based recommendation and solves the problem of too sparse data.

### 2.2. Trust-Based CF Recommender Systems

Different from the original collaborative filtering algorithm, in addition to using user-item ratings, trust-based CF recommendation can also further mine the association between users and friends and use it as auxiliary information to generate final recommendations for users. It can be seen that social recommendation can significantly improve the effect of recommendation and alleviate the problem of too sparse data in the system. Golbeck proposes the TidalTrust to extract user preferences through user trust relationship and then generate recommendation [26]. However, the trust information is more sparse than the rating matrix, so this similarity method may cause even severer data sparsity problem. Authors in [27] propose the SocialMF approach that learns the latent feature vectors of users and items. The feature vector of each user especially is dependent on those of his direct neighbors in the social network. Experiments show that this method can improve the accuracy of recommendation while alleviating the user's cold start problem. In view of the sparse data, in order to accurately extract the user's preference factors, some researchers have begun to use trust and other information in the recommendation. Authors in [28] propose a new user similarity method that make use of both ratings and trust. Experiments show that it can alleviate data sparsity and cold start while improving the recommendation accuracy. Bok et al. propose a new recommendation algorithm based on heuristic similarity and trust which can alleviate the problem of data sparsity, cold start, and trust measure [29].

Based on the above research works, aiming at the problem of data sparsity in collaborative filtering algorithm, a recommendation approach based on user interest and trust value is proposed.

## 3. Proposed Approach

**Figure 1:**
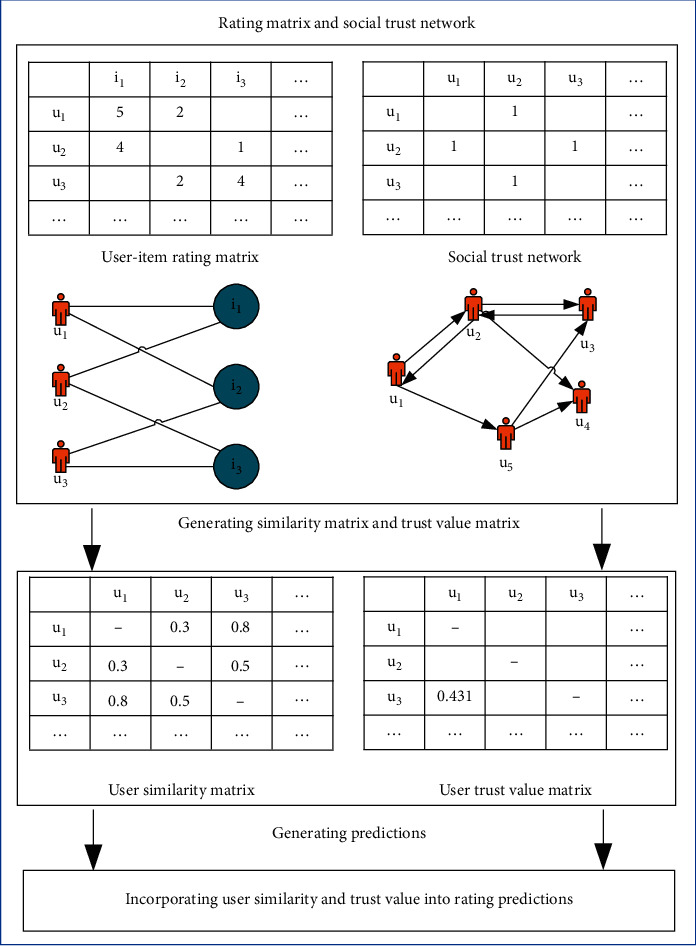
Structure of the proposed algorithm.

In this section, we mainly introduce the steps of the improved algorithm, including (1) calculation of user trust value, (2) calculation of user interest, (3) reconstruction of personalized recommendation weight, and (4) recommendation prediction. The general flow of the improved method is shown in Figure 1. The details of the main steps are described below.

### 3.1. The Improvement of the Calculation of Trust Value

**Figure 2:**
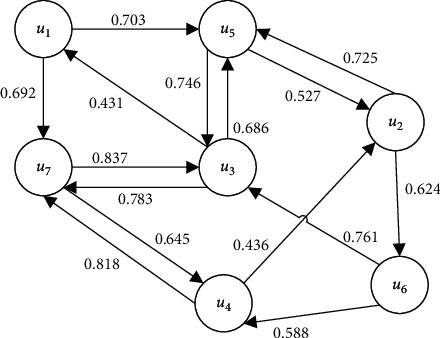
Network of trust between users.

To alleviate the data sparsity problem of recommendation algorithm, social network trust information is used to match accurate neighbor users to target users to improve the recommendation accuracy. In social networks, the attention between users reflects the trust between users, as shown in Figure 2. The arrow indicates that the user is the target trusted user; for example, *u*_1_⟶*u*_5_ indicates that user 1 trusts user 5.

This paper calculates the trust value between users and constructs the trust set Tu based on the attention between users.

Definition 1 of trust network is as follows: for a given social network, we can see a trust network formed by the trust values among users *Q* = (U, *E*, D), where U is the set of users, *E* is the set of directed edges in the trust network, edge e (*u*_i_, *u*_j_) represents the user's trust relationship between user *u*_i_ and user *u*_j_, and *D* represents the set of trust degrees of the directed edge. Let *Ttrust* denote the degree of trust. For example, the user's trust degree to the user is recorded as Ttrust_u0, u1_.

Definition 2 of trust path is as follows: in a given trust network *Q* = (*U*, *E*, *D*), the target user's trust perception of indirect trust user *u*_i_ is based on an accessible path *P* = (*u*_i_,…, *u*_x_, *u*_y_,…, *u*_z_) and the trust value of any edge e (*u*_x_,*u*_y_) on path *P* is greater than the set trust threshold; then path P is a trust path. For example, the transfer path from user *u*_0_ to user *u*_1_ is marked as *P*_01_. However, the trust will also decay with the increase of the path, so we set a certain hop threshold in the trust path.

In network social activities, the trust between users is generally divided into direct trust and indirect trust [30]. Direct trust is the one-to-one trust based on certain cognition between users, while indirect trust is the trust that users place on another user due to the recommendation of a middleman. In general, the trust value between users is represented by a certain value in [0, 1]. The more the trust value approaches 1, the more trust it will be. If the trust value is 0, it means no trust.

#### 3.1.1. The Calculation of the User's Direct Trust

Generally speaking, in social networks, if a user is trusted by more other users, it generally indicates that the user's credibility is high, and vice versa. According to the traditional concept, the trust between users is equivalent. That is, if user *u*_0_ trust user *u*_1_, then user u_1_will also trust user *u*_0_, and the trust degree between the two is equivalent. However, in real life, the degree of trust between user *u*_0_ and user *u*_1_ is usually not equivalent. If user *u*_0_ trusts user *u*_1_, it does not mean that user u_1_ must trust user *u*_0_. Therefore, the trust weight is introduced in this paper, as shown in the following equations:

(1) $$\begin{array}{c} \gamma_{u_{0},u_{1}}=\frac{U\left(u_{0}\right)}{U\left(u_{0}\right)\cup U\left(u_{1}\right)}, \end{array}$$

(2) $$\begin{array}{c} \gamma_{u_{1},u_{0}}=\frac{U\left(u_{1}\right)}{U\left(u_{0}\right)\cup U\left(u_{1}\right)}, \end{array}$$

where *U* (*u*_0_), *U* (*u*_1_) represents the set of user *u*_0_, user *u*_1_ concerned and *U* (*u*_0_)∪*U* (*u*_1_) represents the number of user set concerned by user *u*_0_ or *u*_1_. Equations (1) and (2) calculate the trust weight of user *u*_0_ to user *u*_1_ and the trust weights of user *u*_1_ to user *u*_0_.

In Definition 3, Dtrust represents direct trust. According to the user relationship matrix *T*, for any *u*_0_, *u*_1_, if the user *u*_0_ pays attention to the user *u*_1_, there is Dtrust_u0, u1_, denoted as *u*_0_⟶*u*_1_.

The calculation formula of direct trust is shown in the following formulas:

(3) $$\begin{array}{c} {\text{Dtrust}}_{u_{0},u_{1}}=\frac{U\left(u_{0}\right)}{\left|U\left(u_{0}\right)\cup U\left(u_{1}\right)\right|}*\frac{\left|U\left(u_{0}\right)\cap U\left(u_{1}\right)\right|}{\left|U\left(u_{0}\right)\cup U\left(u_{1}\right)\right|}, \end{array}$$

(4) $$\begin{array}{c} {\text{Dtrust}}_{u_{1},u_{0}}=\frac{U\left(u_{1}\right)}{\left|U\left(u_{0}\right)\cup U\left(u_{1}\right)\right|}*\frac{\left|U\left(u_{0}\right)\cap U\left(u_{1}\right)\right|}{\left|U\left(u_{0}\right)\cup U\left(u_{1}\right)\right|}, \end{array}$$

where |*U* (*u*_0_)∪*U* (*u*_1_)| represents the number of sets of users that user *u*_0_ and user *u*_1_ are concerned about together.

#### 3.1.2. The Calculation of the User's Indirect Trust

In Figure 2, the trust between user nodes is generated based on the direct trust of users face to face. However, in the actual social network, there may be no or no obvious potential trust relationship between many users. As a result, the trust matrix is very sparse, and it will be much more difficult to calculate the trust similarity. Therefore, to match more neighbor users to target users, this paper introduces the trust transfer property of social network to calculate the trust degree between users without intersection, to connect more users who are not directly associated with each other.

In Definition 4, Itrust represents indirect trust; if *u* trusts *w* and *w* trusts *v*, then there is Itrust_*u, v*_; otherwise, there is no indirect trust relationship.

In this paper, before calculating the user trust matrix, trust transfer is introduced to calculate the trust between users without intersection. If there is no direct trust relationship between two users, the calculation formula of their trust degree is as follows:

(5) $$\begin{array}{c} {\text{Itrust}}_{u_{i},u_{j}}=\frac{\sum_{k=1}^{n}\left(W\left(k\right)\times W_{\text{direct}}^{k}\right)}{\sum_{k=1}^{n}W\left(k\right)}, \end{array}$$

where Dtrust_ui, uj_ represents the user *u*_i_'s indirect trust to the user *u*_j_, *W*^*k*^_direct_ represents the trust value before the user *u*_i_ reaches the user u_j_ in the trust path of *k*, and *W* (*k*) represents the weight of the *k*th trust path. The calculation formula is as follows:

(6) $$\begin{array}{c} W\left(k\right)=\prod_{i=1}^{l-1}{\text{Dtrust}}_{i}\left(x,y\right), \end{array}$$

*l* represents the length of the k-th relationship path, and Dtrust*_i_* (*x, y*) represents the direct trust between users *u_x_* and *u_y_* in the k-th path.

According to the six degrees of separation theory discovered by Stanley Milgram [31], the trust threshold is set as *W*_*θ*_ = 0.5 and *h*_*θ*_ = 6. As shown in Figure 2, if we calculate the user *u*_2_'s indirect trust to the user *u*_7_, there are five paths: *u*_2_⟶*u*_6_⟶*u*_4_⟶*u*_7,_*u*_2_⟶*u*_6_⟶*u*_3_⟶*u*_7,_*u*_2_⟶*u*_5_⟶*u*_3_⟶*u*_7_, *u*_2_⟶*u*_6_⟶*u*_3_⟶*u*_1_⟶*u*_7_, and *u*_2_⟶*u*_5_⟶*u*_3_ ⟶*u*_1_⟶*u*_7_. Then, the trust path from user *u*_2_ to user *u*_7_ is *P*_2647_, *P*_2637_, *P*_2537_, *P*_25317_, and *P*_26317_. According to (6), *W* (1) = 0.624 *∗* 0.588 = 0.3669, *W* (2) = 0.624 *∗* 0.761 = 0.4749, *W* (3) = 0.725 *∗* 0.746 = 0.5409, *W* (4) = 0.725 *∗* 0.746 *∗* 0.431 = 0.2331, and *W* (5) = 0.624 *∗* 0.761 *∗* 0.431 = 0.2047. According to (5), the indirect trust degree of user *u*_2_ to user *u*_7_ can be obtained as follows:

(7) $$\begin{array}{c} {\text{Itrust}}_{u_{2},u_{7}}=\frac{W\left(1\right)*0.818+W\left(2\right)*0.783+W\left(3\right)*0.783+W\left(4\right)*0.692+W\left(5\right)*0.692}{W\left(1\right)+W\left(2\right)+W\left(3\right)+W\left(4\right)+W\left(5\right)}=0.768. \end{array}$$

The total trust of users is obtained by integrating the direct trust Dtrust_*u,v*_ and indirect trust Itrust_*u,v*_ of users, which will be taken as the similarity value of user trust Trust_u,v_, as shown in the following:

(8) $$\begin{array}{c} {\text{Trust}}_{u,v}=\left\{\begin{array}{cc} {\text{Dtrust}}_{u,v}, & {\text{Dtrust}}_{u,v}\neq 0,\\ {\text{Itrust}}_{u,v}, & {\text{Dtrust}}_{u,v}=0,{\text{Itrust}}_{u,v}\neq 0,\\ 0, & {\text{Dtrust}}_{u,v}=0,{\text{Itrust}}_{u,v}=0, \end{array}\right) \end{array}$$

where if Dtrust_*u,v*_ is not zero, Dtrust_*u,v*_ means the direct trust value of users; when Dtrust_u,v_ = 0, if Itrust_u,v_ is not zero, it means that there is only an indirect trust value between users; when Dtrust_*u,v*_ and Itrust_*u,v*_ are both zero, it means that there is no trust between users.

The user trust matrix is established based on the above trust values, which is represented by Trust_*m×m*_ here:

(9) $$\begin{array}{c} {\text{Trust}}_{m\times m}=\left[\begin{array}{cccc} {\text{Trust}}_{u_{1},v_{1}} & {\text{Trust}}_{u_{1},v_{2}} & \cdots & {\text{Trust}}_{u_{1},v_{m}}\\ {\text{Trust}}_{u_{2},v_{1}} & {\text{Trust}}_{u_{2},v_{2}} & \cdots & {\text{Trust}}_{u_{2},v_{m}}\\ \cdots & \cdots & \cdots & \cdots \\ {\text{Trust}}_{u_{m},v_{1}} & {\text{Trust}}_{u_{m},v_{2}} & \cdots & {\text{Trust}}_{u_{m},v_{m}} \end{array}\right], \end{array}$$

where Trust_*um,vm*_ is the user *u*_m_'s trust to the user *v*_*m*_.

### 3.2. The Improvement of the Calculation of Interest Value

Similarity measurement is essential for collaborative filtering recommendations. A good similarity measurement method can affect the accuracy of recommendation results. Pearson similarity is a widely used similarity calculation method. It is used to average the variables, which can reduce the influence of the numerical difference of individual variables on the similarity between variables, and the value range is - 1–1. It is defined as follows:

(10) $$\begin{array}{c} \text{sim}\left(u,v\right)=\frac{\sum_{i\in I_{uv}}\left(G_{u,i}-\bar{G_{u}}\right)\times \left(G_{v,i}-\bar{G_{v}}\right)}{\sqrt{\sum_{i\in I_{uv}}{\left(G_{u,i}-\bar{G_{u}}\right)}^{2}}\sqrt{\sum_{i\in I_{uv}}{\left(G_{v,i}-\bar{G_{v}}\right)}^{2}}}. \end{array}$$

However, the original Pearson similarity is highly dependent on the common score, which often ignores the items that have no common score among users. When the data is sparse, there are very few or no common scoring items that can be used, which will affect the accuracy of the recommendation. In fact, those discrete noncorating data also carry user preference information, so we should use this information as much as possible. Bhattacharyya coefficient [32] is a similarity measurement method, which can be used to calculate the correlation between the scores of noncommon items between two users.

Suppose that in the discrete domain *z* is all score values, the number of scoring items of item *i* is *n*_i_, the number of scoring items of item *j* is *n*_j_, and the probability density distribution of two items with the score of *h* is ${\hat{p}}_{ih}$ and ${\hat{p}}_{jh}$, where ${\hat{p}}_{ih}=\#h/n_{i}$ and ${\hat{p}}_{jh}=\#h/n_{j}$. #h is the number of items *i* and *j* scoring *h*; then BC similarity of item *i* and item *j* is expressed as follows:

(11) S=itemBCc, d=BCp^c, p^d=∑x=1zp^cxp^dx.

According to the above analysis, this paper intends to use item similarity to modify the user similarity weighted, and the formula is as follows:

(12) $$\begin{array}{c} s_{\text{userp}}\left(c,d\right)=\sum_{i\in I_{c}}\sum_{j\in I_{d}}S_{\text{item}}\cdot S\left(g_{ci},g_{dj}\right). \end{array}$$

In (12), user similarity is calculated by Pearson similarity as (10), and item similarity is calculated by (11).

### 3.3. Rating Prediction Based on the Relationship between User Interest and Trust

According to the relationship between user interest and trust, by setting the weight *δ* to balance the weight of user interest and trust, the ability to identify neighbors can be improved. In different applications, the degree of dependence on the two kinds of information is different. By adjusting the value of *δ*, the influence of the two kinds of information on the prediction rating can be adjusted to avoid the problem of large weight of interest or trust.

(13) $$\begin{array}{c} {\text{Sim}}_{u,v}=\left\{\begin{array}{cc} {\text{Trust}}_{\text{u}\times v}, & \delta =0,\\ \delta {\text{sim}}_{g\left(u,v\right)}+\left(1-\delta \right){\text{Trust}}_{\text{u}\times v}, & 0<\delta <1,\\ {\text{sim}}_{\text{g}\left(u,v\right)}, & \delta =1. \end{array}\right) \end{array}$$

When *δ* = 0, Sim_*u, v*_ only represents the user's interest similarity value. When *δ* = 1, Sim_*u, v*_ only represents the user's trust similarity value; when 0 < *δ* < 1, Sim_*u, v*_ represents the comprehensive user interest similarity value and trust similarity value.

According to the steps of collaborative filtering recommendation, some users with the highest comprehensive similarity are regarded as the neighbor set *N* (*u*) of the target user *u*, and the users in the set are ranked from high to low according to their similarity with *u*. According to the similar neighbors, predicting the score of user *u* on the unrated item *i* is as follows:

(14) $$\begin{array}{c} P_{u,i}={\bar{r}}_{u}+\frac{\sum_{v\in N\left(u\right)}\left(r_{v,i}-{\bar{r}}_{v}\right)*{\text{sim}}_{u,v}}{\sum_{v\in N\left(u\right)}{\text{sim}}_{u,v}}, \end{array}$$

where ${\bar{\text{r}}}_{u}$ and ${\bar{r}}_{v}$, respectively, represent the average score of user *u* and user *v* for the item.

## 4. Experiment Results and Discussion

### 4.1. Experiment Datasets

In this paper, we use the FilmTrust dataset. It can be divided into two parts: rating set and trust set. The rating set contains 35,497 item ratings from 1508 users who have rated at least once among 2071 different items. The rating takes values in the range [0.5–4.0] with an increment of 0.5. The trust set contains 1853 trust statements that are given by 609 trustor users to 732 trustee users. If the user trust another, then the trust value of the user to another is 1; otherwise, the value is 0. The specific rating information and trust information of the dataset are shown in Table 1. For the rating set, the proposed algorithm is trained according to the division of 80% training set and 20% test set.

### 4.2. Performance Metrics

In this experiment, the dataset is divided into two parts according to the proportion of 80% and 20%. The former is used as the training set to construct the recommendation model, while the latter is used as the test set. In this experiment, mean absolute error (MAE) and root mean square error (RMSE) were used to measure the accuracy of the recommendation results by calculating the error between the real score and the predicted score. The smaller the difference, the smaller the deviation, and the better the recommendation effect, as shown in the following:

(15) $$\begin{array}{c} \text{MAE}=\frac{1}{N}\left(\sum_{u,i}\left|r_{u,i}-p_{u,i}\right|\right), \end{array}$$

(16) $$\begin{array}{c} \text{RMSE}=\sqrt{\frac{1}{N}\sum_{u,i}{\left(r_{u,i}-p_{u,i}\right)}^{2}}, \end{array}$$

where *r*_u, i_ is the predicted score of candidate item *i*, *p*_u, i_ is the actual score of item *i*, and *N* is the number of candidate items.

### 4.3. Results of Evaluation

#### 4.3.1. Choice of *δ*

**Figure 3:**
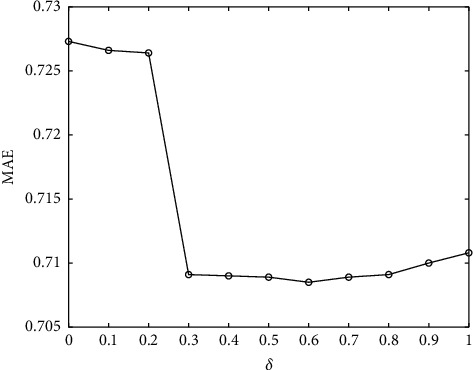
The influence of parameter *δ* on MAE.

**Figure 4:**
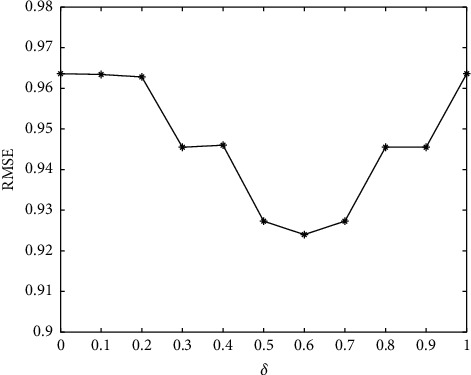
The influence of parameter *δ* on RMSE.

In this paper, the similarity calculation is composed of two parts: user's interest and trust. *δ* is used as the adjustment factor, the value is [0, 1], and the interval is 0.1. Figures 3 and 4 show the change trend of *δ*. With the increase of *δ*, MAE and RMSE decrease at first and then increase, indicating that the different proportions of interest and trust will directly affect the recommendation results of the algorithm. From the figure, we can get the minimum value of MAE and RMSE at *δ* = 0.6 on the FilmTrust dataset. Considering the MAE and RMSE values of interest and trust, when *δ* = 0.6, the algorithm has the best recommendation results.

#### 4.3.2. Comparison of Recommendation Performance of Several Algorithms

To comparatively evaluate the performance of our method, we select the following representative models as comparison methods, including UBCF [33], SVD++ [34], and TrustSVD [22].

**Figure 5:**
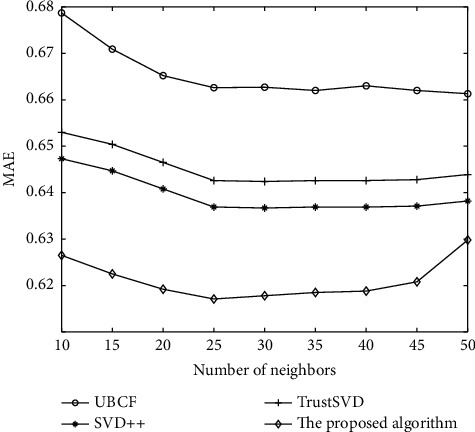
Comparison of recommendation performance of several algorithms on MAE.

**Figure 6:**
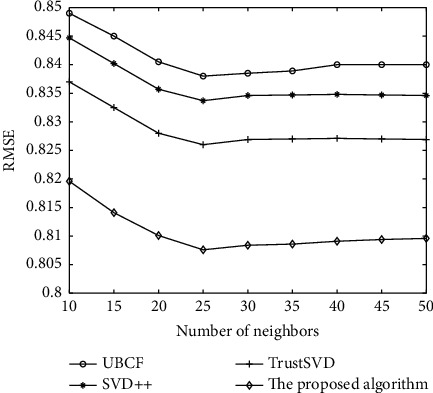
Comparison of recommendation performance of several algorithms on RMSE.

As shown in Figures 5 and 6, the comparison results of each algorithm on the FilmTrust dataset are shown. It can be analyzed from the experimental result diagram that MAE and RMSE values of UBCF algorithm on the FilmTrust dataset are much higher than those of the other three algorithm models, indicating that other algorithm models can effectively improve the recommendation accuracy of the recommendation algorithm. On the FilmTrust dataset, through the horizontal comparative analysis of the experimental results, the MAE and RMSE values of SVD++ algorithm, TrustSVD algorithm, and the algorithm in this paper are the minimum values when the neighbour's number is *N* = 25. Then, with the increase of the number of neighbors, MAE and RMSE values will not decrease again and slightly increase, which shows that as recommended, increase in the number of each algorithm of MAE and RMSE value will not drop, and the dataset's best neighbour's number is 25. The results show that the MAE and RMSE values of the proposed algorithm are smaller than those of other algorithms on the FilmTrust dataset, which proves that the proposed algorithm has a good effect on improving the recommendation accuracy.

In order to understand the comparison effect of the experiment more intuitively, this paper presents the experimental results in the form of a table.  Table 2 shows the MAE and RMSE values of the improved algorithm when the number of nearest neighbors is different. It can be seen from the table that as the number of nearest neighbors N changes, the values of MAE and RMSE both show a trend of first decreasing and then increasing. Only when *N* = 25, these two indicators achieve the minimum value.

According to the conclusions obtained from the previous analysis, Table 3 shows the values of MAE and RMSE corresponding to different algorithms with *N* = 25. Through observation, it can be known that the values of the two indexes of SVD++, TrustSVD and the algorithm in this chapter are lower than those of the UBCF algorithm. Compared with the UBCF algorithm, the MAE values of SVD++, TrustSVD, and the algorithm in this chapter are reduced by 3.88%, 3.02%, and 6.87%, respectively. Compared with the UBCF algorithm, the RMSE values of SVD++, TrustSVD, and the algorithm in this chapter are reduced by 0.51%, 1.43%, and 3.63%, respectively. It can be seen that these three algorithms have improved the recommendation effect compared with the UBCF algorithm, and the improved algorithm in this paper has a better recommendation effect.

## 5. Conclusion

Aiming at the deficiency of the traditional collaborative filtering recommendation algorithm in the case of sparse data, this paper proposes an improved collaborative filtering recommendation algorithm that integrates the user's trust value and interest. The improved similarity method is used to calculate the interest similarity between users. Combined with the trust relationship of social network, the user trust set is established by calculating the direct trust value and the indirect trust value. Finally, the neighbor users are selected based on the user's interest and trust set, and recommendations are generated for the user according to the neighbor set. The algorithm alleviates the data sparsity problem and improves the recommendation efficiency and accuracy of the system. In future work, we consider using matrix factorization techniques to further improve the quality of recommendation.

## Figures and Tables

**Table 1:** Statistics of the dataset.

Dataset	#Users	#Items	#Ratings	#Trusts	Rating density (%)	Trust desity (%)
FilmTrust	1508	2071	35497	1853	1.14	0.08

**Table 2:** MAE and RMSE value of the proposed algorithm.

Evaluation metrics	10	15	20	25	30	35	40	45	50
MAE	0.6265	0.6225	0.6192	0.6171	0.6178	0.6185	0.6188	0.6208	0.6298
RMSE	0.8196	0.8141	0.8101	0.8076	0.8084	0.8086	0.8091	0.8094	0.8096

**Table 3:** MAE and RMSE value of several algorithms.

Evaluation metrics	UBCF	SVD++	TrustSVD	The proposed algorithm
MAE	0.6626	0.6369	0.6426	0.6171
RMSE	0.8380	0.8337	0.8260	0.8076

## Data Availability

In this paper, the FilmTrust dataset has been used (https://github.com/Coder-Yu/SDLib/tree/6645c9723fd3f8fe6fd8699eaf88b71874df719c/dataset/filmtrust).
